# Amperometric Detection of Conformational Change of Proteins Using Immobilized-Liposome Sensor System

**DOI:** 10.3390/s18010136

**Published:** 2018-01-05

**Authors:** Hyunjong Yu, Young Hwan Son, Hak-Jin Kim, Keesung Kim, Pahn-Shick Chang, Ho-Sup Jung

**Affiliations:** 1 Department of Agricultural Biotechnology, Seoul National University, Seoul 08826, Korea; yhj7654@snu.ac.kr; 2 Department of Rural System Engineering, Seoul National University, Seoul 08826, Korea; syh86@snu.ac.kr; 3 Department of Biosystems Engineering and Biomaterials Science, Seoul National University, Seoul 08826, Korea; kimhj69@snu.ac.kr; 4 Research Institute of Advanced Materials, Collage of Engineering, Seoul National University, Seoul 028826, Korea; keesung@snu.ac.kr; 5 Research Institute of Agriculture and Life Sciences, Seoul National University, Seoul 08826, Korea

**Keywords:** immobilized-liposome, TM-AFM, amperometric sensor, conformational change protein, array system, pattern recognition

## Abstract

An immobilized liposome electrode (ILE)-based sensor was developed to quantify conformational changes of the proteins under various stress conditions. The ILE surface was characterized by using a tapping-mode atomic force microscopy (TM-AFM) to confirm surface immobilization of liposome. The uniform layer of liposome was formed on the electrode. The current deviations generated based on the status of the proteins under different stress were then measured. Bovine carbonic anhydrase (CAB) and lysozyme were tested with three different conditions: native, reduced and partially denatured. For both proteins, a linear dynamic range formed between denatured concentrations and output electric current signals was able to quantify conformational changes of the proteins. The pattern recognition (PARC) technique was integrated with ILE-based sensor to perform data analysis and provided an effective method to improve the prediction of protein structural changes. The ILE-based stress sensor showed potential of leveraging the amperometric technique to manifest activity of proteins based on various external conditions.

## 1. Introduction

Given that interest to fast, reliable and continuous measurements of biological molecules has been rising in medicine, biotechnology and environmental sciences, a biosensor which facilitates point-of-use with high sensitivity and affordability has been widely recognized as one of the promising alternatives in life science [1,2,3]. In synthesizing a biosensor membrane, conducting polymers, enzymes, or other biomaterials have been generally used [4,5,6]. In such various biocompatible materials, lipid bilayer vesicles (liposomes) which are highly structured-supramolecules and analogs to microbial cell membranes, are versatile sensing materials. Liposomes have been used as carriers for a target-specific drug delivery system [3,7,8] or agents for the encapsulation of assay reagents in immunoassays detecting small molecules [9,10,11]. In addition, our previous studies that liposome membranes can sensitively recognize the environmental stresses (heat, pH, salt concentration, osmotic pressure and shear stress) as well as microbial cell membrane functions [12].

The environmental or external stresses exposed to proteins induce the structural deformation of the proteins (secondary and tertiary structure) which can be detected by surface hydrophobicity: surface net hydrophobicity (HFS) and local hydrophobicity (LH). Under stressed states, it was demonstrated that the partially denatured protein in a molten globule (MG) state is a key component for interacting with the membranes [13,14,15]. The protein at MG state has showed a similar secondary structure as the native state but its tertiary structure is not closely packed, resulting in relatively high local hydrophobicity and structural fluctuation at native state [16,17,18]. Recently, some studies on the stress-responsive bioprocess, such as stress-mediated refolding [12] and separation of proteins [19], have been reported. However, the method that quantifies the stress-responsive process using a liposome has not been developed yet. Although there are several methods including array-type protein chip [20], florescence intensity [21] and circular dichroism (CD) [22] measurements to monitor stress-induced structural changes of proteins, those are expensive and labor-consuming. In addition, those methods show the limitations for predicting the dynamics, structure deformation or interaction of proteins.

In this study, a stress sensor for the dynamic analysis (and static analysis) of conformational changes of proteins under various stress conditions was developed by using immobilized liposome electrode (ILE). Liposomes are spherical phospholipids bilayer vesicles containing an inner micro-water pool and can entrap almost any water-soluble (marker) molecules within the interior of those lipid vesicles. In general liposome-based sensors, the liposomes are disrupted by either complement lysis or surfactant lysis [23]. Upon the process of the membrane fluctuation, the entrapped markers are released and can be detected by an appropriate transducer owing to the increase of the membrane motion. Therefore, the measurement of the markers can be used as a secondary response for monitoring protein-lipid membrane interactions, which are primary detection scheme used for the ILE-based sensor. To achieve aforementioned prediction of protein structural changes, we describe the analytical characteristics of the resultant amperometric ILE-based sensor due to the electrolyte release from liposomes in the presence of proteins under various denaturant concentrations. As a result, we finally analyzed the response of ILE to proteins with pattern recognition (PARC) to improve the prediction of protein structural changes.

## 2. Materials and Methods

### 2.1. Materials

1-Palmitoyl-2-oleoyl-*sn*-glycero-3-phosphocholine (POPC) and egg yolk phosphatidyl-ethanolamine (EPE) were purchased from Avanti Polar Lipids (Alabaster, AL, USA). Chitosan-hexamer was purchased from Seikagaku Co. (Tokyo, Japan). Bovine carbonic anhydrase (CAB) and 16-mercaptohexadecanoic acid were obtained from Sigma (St. Louis, MO, USA). Hen egg white lysozyme, cholesterol, guanidinium hydrochloride (GuHCl), dithiothreitol (DTT), *N*-hydroxysuccinimide (NHS), dioxane and potassium hexacyano-ferrate (II) were purchased from Wako (Osaka, Japan). 1-ethyl-3-(3-dimethlaminopropyl)-carbodiimide hydrochloride (WSC) and 1-(4-trimethyl -ammoniumphenyl)-6-diphenyl-1,3,5-hexatriene (TMA-DPH) were purchased from Dojindo Laboratories (Kumamoto, Japan). All other chemicals used were of analytical reagent grade.

### 2.2. Preparation of Electrolyte-Encapsulated Liposomes

POPC liposomes were prepared by previously described methods [24]. POPC (9.8 mg/mL) and EPE (0.2 mg/mL) dissolved in chloroform were dried in a 100 mL round-bottom flask by rotary evaporation under reduced pressure. The lipid was redissolved in diethyl ether twice and then the solvent was evaporated again. The lipid film was kept under high vacuum for at least 3 h and then hydrated with 50 mM potassium hexacyano-ferrate (II) solution in 50 mM phosphate buffer (pH 7.5) at room temperature to form multilamellar vesicles (MLVs). The vesicle suspension was frozen (−80 °C) and thawed (37 °C) to enhance the transformation of small vesicles into larger MLVs. Freezing-thawing cycles were applied 5 times before forcing the liposome solution 15 times through a polycarbonate membrane (pore diameter; 100 nm) using an extruder device (Liposofast; Avanti Polar Lipid, Inc., Alabaster, AL, USA). Unencapsulated potassium hexacyano-ferrate (II) was removed from the liposome sample by size exclusion chromatography on a Sepharose CL-4B column with 100 mM Tris-HCl buffer as the mobile phase. The liposomes were stored at 4 °C before use.

For the preparation of chitosan-coated liposome (POPC/CHT), chitosan-hexamer was dissolved in phosphate buffer to form a 0.005% (*w*/*v*) solution. Liposome suspension was added dropwise into the chitosan solution under stirring in a volume ratio 1:4 [25]. The suspension was left overnight at 4 °C to stabilize prior to characterization. Chitosan-coated liposome were harvested from the mixture by centrifugation at 57,400× *g* for 20 min at 4 °C and resuspended in potassium hexacyano-ferrate (II) buffer solution. The POPC/cholesterol33% (POPC/CH33) electrode was modified with POPC (6.5 mg/mL), EPE (2 mg/mL) and cholesterol (3.3 mg/mL) dissolved in chloroform solution. The other operations were followed by the above preparation method.

### 2.3. Tapping-Mode Atomic Force Microscopy (TM-AFM)

All TM-AFM images were acquired using Digital Instruments (DI) Nanoscope IIIA Multimode scanning probe microscope (Digital Instruments, Santa Barbara, CA, USA) with 120 mm oxide-sharpened silicon nitride V-shaped cantilevers. The TM-AFM images were acquired using the E scanning head, which has a maximum lateral scan area of 14.9 ×14.9 µm. The imaging was performed at 1 to 2 Hz of tip scan rates and 337 kHz of the cantilever drive frequencies at room temperature. The images were captured at 512 × 512 pixel and were low-pass filtered. Feature size and volumes were calculated using the DI Nanoscope software (version 4.22r2). 

### 2.4. Preparation of Immobilized Liposome Electrode (ILE)

**Scheme 1:**
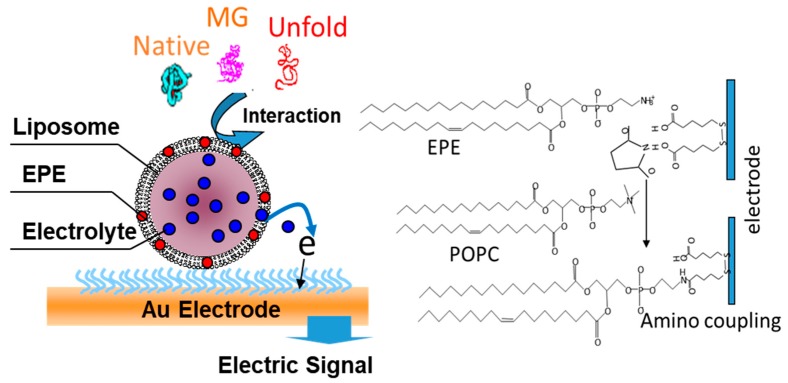
Schematic illustration of amino coupling process using EPE/POPC liposome on SAM layers onto gold (Au) electrode surface and showing to produce the electric signal due to the electrolyte release from liposome membrane according to protein structural as like as native, molten globule (MG) and unfold (denature) states.

To fabricate the ILE, a self-assembled monolayer (SAM) using 16-mercaptohexadecanoic acid was formed on a gold (Au) electrode (2 × 2 mm). The electrode was immersed into a solution consisting of dioxane: deionized (DI) water (90:10), 17 mM NHS and 17 mM WSC for 4 h in order to being pre-activated. The liposome was then immobilized on the SAM layer by the amino conjugated method. After 1 h, the liposome immobilized on the Au electrode was rinsed with phosphate buffer (pH 7.5) several times. The immobilized liposome was shown in Scheme 1. The ILE was used to measure the electric response against various states of protein structures under denaturant concentrations.

### 2.5. Electrochemical Measurement

A potentiostat/galvanostat (EG&G model 273) connected to a personal computer (EG&G Software Power suit #270/250) were used for the electrochemical measurements. A three-electrode cell: (1) the Au electrode modified with liposome (working electrode); (2) the platinum wire (counter electrode) and (3) the Ag/AgCl was used. Cyclic voltammetry (CV) and chronoamperometry (CA) were performed in 50 mM phosphate buffer (pH 7.5) containing 1 mM Na_2_SO_4_ as a background electrolyte solution at 100 mVs^−1^ of the scan rate and a polarized +290 mV (versus Ag/AgCl), respectively.

### 2.6. Membrane Permeability

The membrane permeability of the liposome was determined by using encapsulated calcein liposome. Lipid film was hydrated with Tris-HCl buffer (pH 7.5) containing 100 mM calcein. Unencapsulated calcein was removed by gel permeation chromatography (Sepharose 4B). Otherwise, the method used for synthesizing the liposome was identical. The released fluorescent intensity (RF) was measured with fluorescence detector (FP-777 JASCO Co. Ltd., Tokyo, Japan) (λ_ex_ = 490 nm, λ_em_ = 520 nm).

## 3. Results and Discussion

### 3.1. Characterization of ILE

A homogeneous layer of liposome was formed on the sensor surface with high stability for the detection of the stress-induced structural changes of proteins. After the SAMs were prepared on Au electrode, the liposomes were expected to be immobilized on the surface based on amino coupling.

**Figure 1:**
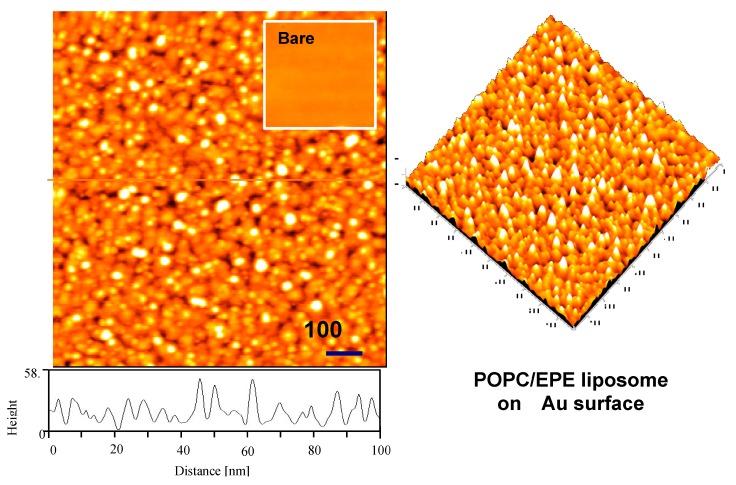
TM-AFM topographic images of immobilized liposome layer on Au electrode surface which was treated with self-assembly layer (SAMs) of HSCH_2_ (CH_2_)_14_COOH for amino coupling conjugation between liposome and SAMs. The bare Au electrode surface are shown in the inset.

The physical morphology of bare Au electrode surface is shown in the inset in Figure 1. The Au electrode has a flat surface and the average height roughness is approximately 2 nm. Figure 1 shows that the TM-AFM images recorded after immobilization of liposomes onto the activated SAMs layers. The liposome size and height, though the variation is slightly smaller than original size, are confirmed as approximately 30~50 nm and 40 nm, respectively. Because the buffer solution of liposomes is dried out since TM-AFM image process in air condition. Uniform layer of liposomes was produced. From our previous studies, the uniform layer of the liposome significantly enhanced the permeability of the liposome membrane if the protein with stress-induced structural changes approached close to the membrane. After the surface of electrode was modified with carboxyl groups, rinsing the liposome-immobilized Au surface was critical to produce evenly distributed coating surface. In the above experiments, it was revealed that the liposome layers immobilized on SAMs were stable for two weeks at room temperature.

Given that the electrochemical reaction such as dissolved redox ions could be substantially suppressed by the coexistence of organic layers, potassium hexacyano-ferrate (II) could be enclosed successfully in the liposome which has well defined redox characteristics and low oxidation potential minimizing interference by other oxidizable molecules in the media.

**Figure 2:**
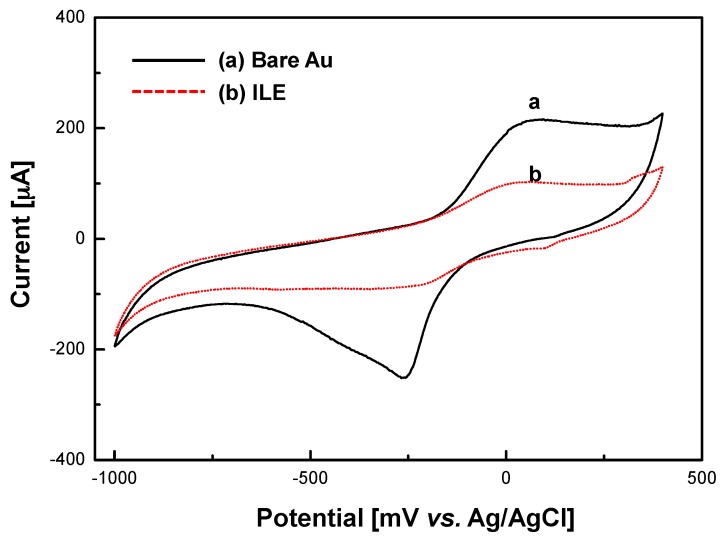
Electrochemical measurements (cyclic voltammogram) onto bare Au electrode (curve a, solid line) and immobilized-liposome electrode (curve b, dot line) were carried out in 1 mM Na_2_SO_4_ as a background electrolyte solution at 100 mVs^−1^ of the scan rate.

Figure 2 shows that CV curves of the redox probe, solubilized in electrolyte solution, on a bare Au electrode (curve a) and on the subsequently formed ILE (curve b). The curve of bare Au electrode is typical for a diffusion controlled reversible redox process. The amperometrical responses of both electrodes were quite different. The immobilization of POPC including EPE liposome on the Au electrode resulted in a significant decrease in amperometrical response compared with bare Au electrode. The large decrease in the amperometrical response indicated that the interfacial electron-transfer between the redox probe and the gold surface was mostly blocked with liposome layers.

**Figure 3:**
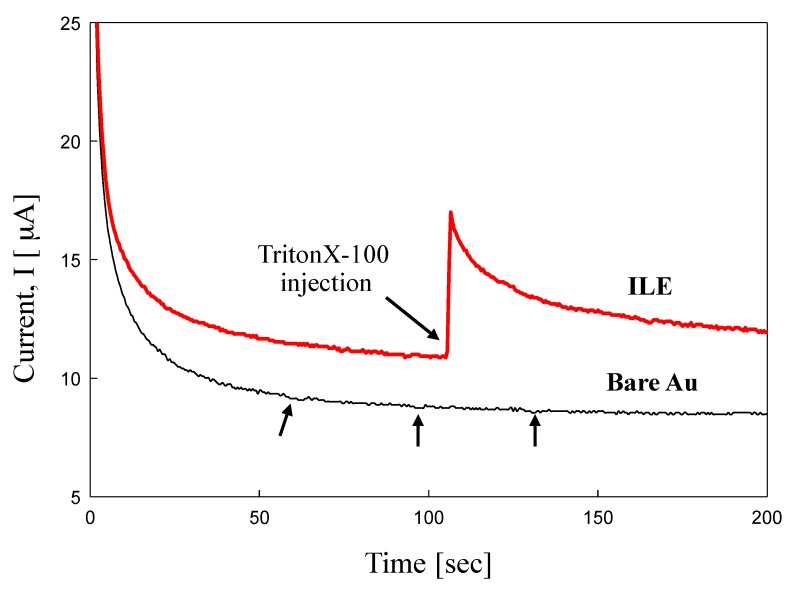
Chronoamperometric measurement carried out in 50 mM phosphate buffer solution using bare Au and immobilized-liposome electrode (ILE). For perturbation of membrane, 5 mM tritonX-100 solution is injected on electrochemical measurement solution (black arrows indicate injection point). Detect condition is +290 mV (vs. Ag/AgCl).

In addition, we investigated whether the ILE contained an electrolyte solution within interior of immobilized liposome or not. The electric response for the electrolyte-loaded liposome has been examined amperometrically. The electrolyte-loaded liposomes (10 µL) were coated on Au electrode at 290 mV potential *vs* Ag/AgCl reference electrode prior to the application of 10 µL of 5 mM tritonX-100 as surfactant due to perturbation of membrane. The output current of bare Au and ILE is shown in Figure 3, respectively. A bare Au electrode had no significant current change for surfactant injection repeatedly, while one from electrolyte-loaded liposome Au electrode (ILE) increased immediately upon the injection of tritonX-100 as surfactant.

### 3.2. Response Behavior of Various Composition of ILE

**Figure 4:**
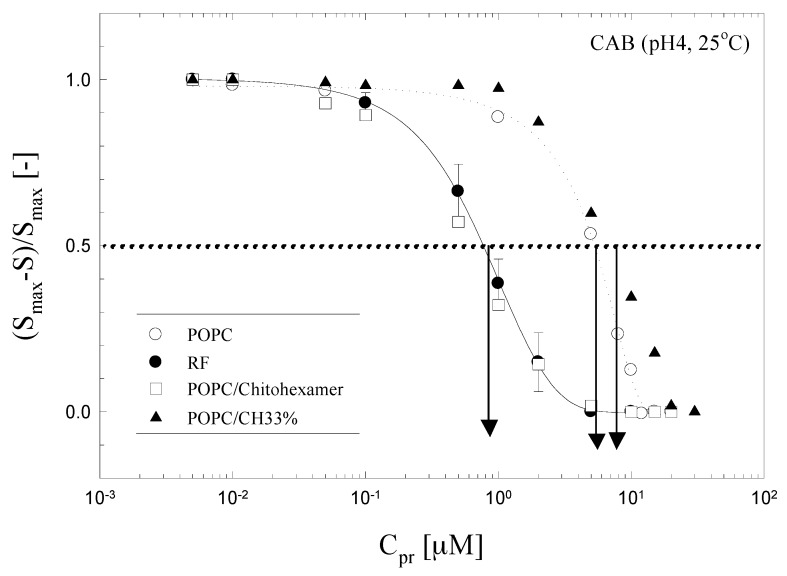
Response behavior of the electrolyte-loaded POPC, POPC/CHT and POPC/CH33 was shown as a function of CAB concentration (pH 4.0) and sensitivity. *S* is the maximum current of the film after injection the CAB; *S_max_* is the maximum current of the film after injection of the saturated concentration of CAB. As a control values, released fluorescent intensity (RF) is obtained the release of calcein from POPC liposome by using the fluorescent measurement.

The electrolyte-loaded liposomes were synthesized on the Au-SAM electrodes by using the liposome with several different compositions, including POPC, POPC/CHT and POPC/CH33. The CAB solution (pH 4.0, 25 °C), which induces a permeability of liposome membrane [25] was applied on the sensor surface in order to test the sensitivity of these electrodes. The response behavior of the electrolyte-loaded POPC, POPC/CHT and POPC/CH33 was shown as a function of CAB concentration (pH 4.0) and sensitivity in Figure 4. In addition, the sensitivity which is defined as (*S_max_ − S*)/*S_max_* was determined under the conditions described in the above experiment (*S* is the maximum current of the film after injection the CAB; *S_max_* is the maximum current of the film after injection of the saturated concentration of CAB). In these experiments, the response behavior of the control system prepared from POPC liposome encapsulating the calcein solution was also investigated.

The sensitivity of POPC and POPC/CH33 electrode was lower than that of control system (RF). The POPC/CHT electrode, however, showed similar sensitivity with that of the control system. Moreover, the signal of the chitosan modified liposome electrode was enhanced at relatively higher magnitude owing to the similar effective permeability against membrane as control where RF was measured with the fluorescence detector. In general, chitosan is a basic polymer with an intrinsic *pK_a_* value of 6.2, of the degree of deacetylation [26]. This suggests that chitosan is protonated and positively charged at neutral pH 4.0 below the *pK_a_* values [27]. Thus, the physically adsorbed chitosan may promote the redox probe to the surface because of the positive charge on chitosan. The chitosan-induced destabilization was also confirmed by a higher initial rate of quinine release from a chitosan-liposome complex compared with the pure liposome [28]. The chitosan caused the disorganization of the ordered bilayer structure and increased fluidity of liposome and sensitivity of sensor film because of the interaction of partially denatured protein.

### 3.3. Response Behavior of the ILE against Proteins Structural Conditions

**Figure 5:**
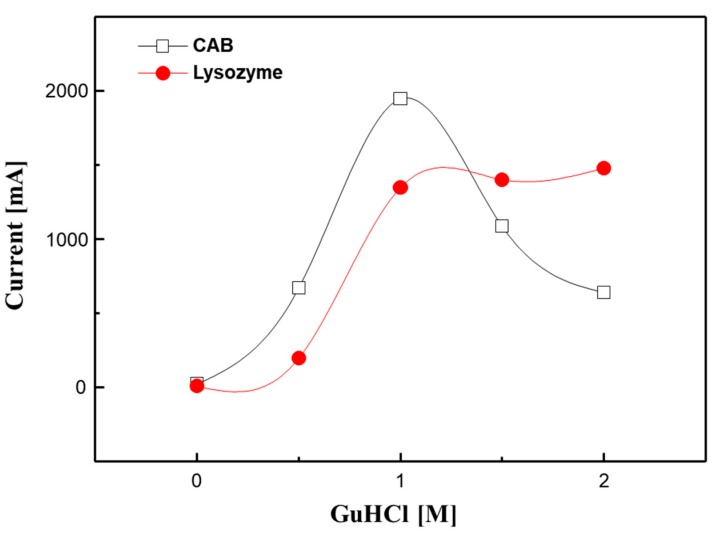
Response electrochemical current behavior of POPC/CHT electrode on the denatured CAB and lysozyme in the presence of GuHCl for denature proteins. Detection condition; +290 mV (vs. Ag/AgCl), 0.05 M phosphate buffer (pH 7.5).

The response behavior of POPC/CHT electrode on the denatured CAB and lysozyme with GuHCl was studied. The output current of electrode upon various conformation of CAB and lysozyme are shown in Figure 5. Conformational change of CAB and lysozyme were controlled by 0.0–2.0 M GuHCl in eluent. Proteins were pre-equilibrated with GuHCl solution for 2 h before applying to the electrode. The output current was increased with increasing the GuHCl concentration up to 1.0 M and then decreased with further increase of GuHCl concentration to 2.0 M. The output current for denatured CAB reached to the maximum value in presence of 1.0 M GuHCl considering that denaturation of CAB begins to occur around 1.0 M GuHCl [29] showing sudden decrease in the biological activity with deformation of tertiary structure [17].

In addition, the response behavior of POPC/CHT electrode on the lysozyme with increased concentration of GuHCl was tested to demonstrate the reliability of the developed method. The output current was increased up to 1.0 M concentration of GuHCl and then reached to the constant value in spite of the increase of the GuHCl concentration up to 2.0 M. It is known that the conformation of lysozyme at the nature state is difficult to be changed to MG state with only treated GuHCl because it has four disulfide bonds to stabilize the tertiary structure. The lysozyme has thus to be treated with 120 mM DTT as reducer for conformational change from native to the MG state.

In the case of the fully unfolded state, its tertiary structure is completely destroyed and the hydrophobic side chains do not associate with each other, which lead to low binding affinity for hydrophobic probes. Although the protein in the MG state maintains the native state of the secondary structure, its tertiary structure is partly destroyed. Therefore, the hydrophobic core of protein is partly exposed to its surface. It has been reported that the proteins only at the MG state can interact with lipid membranes from the immobilized-liposome chromatography [29] and a fluorescence probe leakage experiment [30]. 

Given that proteins only at the MG state can interact with lipid membranes as well as molecular chaperones the partially denatured CAB is effectively interacted with liposomal bilayer membranes through the hydrophobic interaction. In addition, the membrane permeability of liposome increased because of the induction of the perturbation or fluctuation of membrane structures when the protein of the MG state interacts with membrane surface. Thus, an electrolyte-loaded liposome electrode could be used as a protein sensor device with a high sensitivity to detect the state of the conformational changes of proteins.

### 3.4. Principle Component Analysis for Discrimination of Proteins and Its Conditions

The immobilized liposome sensor arrays coupled with pattern recognition (PARC) technique, were used for the analysis of the conformational state of protein under the stress conditions. The results showed that the output current of sensor layers was considerably varied depending on the properties of membrane.

**Figure 6:**
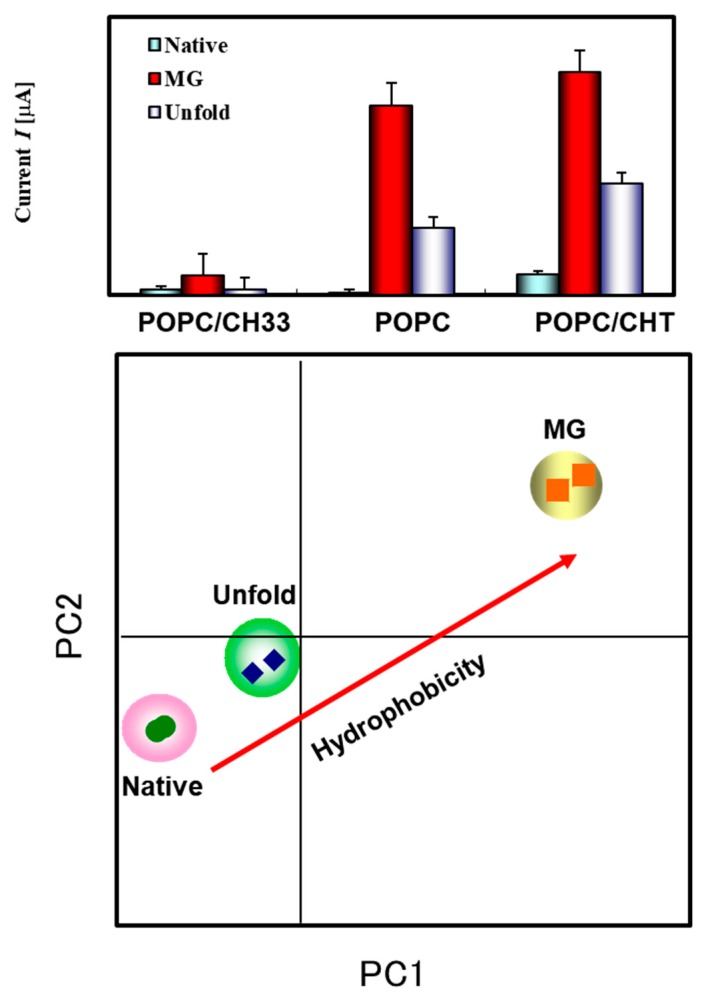
Results of principle of component analysis as pattern recognition technique applied to response patterns using the electrical current signal from proteins structural conditions (native, MG and unfold states) and three types of ILE (POPC, POPC/CHT and POPC/CH33). The used proteins are CAB and lysozyme.

The data points plotted on scatter diagram which were obtained by principal component analysis: multi-variety analysis to reduce the dimensions of data without losing information. In the present case, a nine-dimensional space that was made from three types of membrane (POPC, POPC/CHT and POPC/CH33) and three states of protein condition (i.e., native, MG and unfold states) was reduced to a two-dimensional plane. Each protein group consists of five replicates of three different protein conditions (native, MG and unfold states). Normalization was applied to the data matrix to transform the data into a more expedient form with size effects removed and variance scaled. The PC1 and PC2 results are responsible for discrimination of each protein conditions within three subgroups (Figure 6). The principle component analysis system, when applied to the different response patterns created by matrix of the data, provided well predicted results for the structural changes of the proteins.

## 4. Conclusions

The stress sensor for conformational analysis of proteins was successfully demonstrated by using the biomimetic membrane (liposome)-based stress sensor. The TM-AFM and CV results showed that the electrolyte loaded liposomes were successfully fabricated on Au electrode surface. The ILE with high stability was successfully development for detection of stress-induced conformational changes of proteins. By using the ILE-based stress-sensor, liposome-protein interactions were quantitatively detected with low sample consumption, short assay time and convenient extension to the multi array. In addition, the conformational changes of the proteins under stress conditions were classified by PARC technique, simply. As a result, the ILE system can easily allow to distinguish the protein structural changes without complex detection methods such as circular dichroism (CD). The ILE-based stress sensor showed potential of leveraging amperometric technique to manifest activity of proteins based on various external conditions. This novel sensor system is expected to be applied to the detection of the interactions between liposomes or damaged proteins although the contribution of the hydrogen bonds should furthermore be clarified.
